# Supplementary material for: Highly Selective CO2 Hydrogenation to Methanol over Complex In/Co Catalysts: Effect of Polymer Frame
Source: Nanomaterials (Basel). 2023 Nov 22;13(23):2996. doi: 10.3390/nano13232996 (PMC10708108; doi:10.3390/nano13232996)
Supplement: Supplementary file 1 [file nanomaterials-13-02996-s001.zip › nanomaterials-2734097-supplementary.pdf]

nanomaterials

Supplementary Materials

Highly Selective CO_2 Hydrogenation to Methanol over Complex In/Co Catalysts: Effect of Polymer Frame

Svetlana A. Sorokina [1], Nina V. Kuchkina [1], Stepan P. Mikhailov [2], Alexander V. Mikhalchenko [1], Alexey V. Bykov [2], Valentin Yu. Doluda [2], Lyudmila M. Bronstein [3,*] and Zinaida B. Shifrina [1,*]

[1] A.N. Nesmeyanov Institute of Organoelement Compounds, Russian Academy of Sciences, 28 Vavilov St., 119991 Moscow, Russia; sorokinas@ineos.ac.ru (S.A.S.); kuchkina@ineos.ac.ru (N.V.K.); koal2702@yandex.ru (A.V.M.)

[2] Department of Biotechnology and Chemistry, Tver State Technical University, 22 A. Nikitina St., 170026 Tver, Russia; stefan.oblivion@mail.ru (S.P.M.); bykovav@yandex.ru (A.V.B.); doludav@yandex.ru (V.Y.D.)

[3] Department of Chemistry, Indiana University, 800 E. Kirkwood Av., Bloomington, IN 47405, USA

* Correspondence: lybronst@indiana.edu (L.M.B.); shifrina@ineos.ac.ru (Z.B.S.)

Table S1. Summary of the catalysts synthesized. All catalysts contain SiO_2-Fe_3O_4-PPP as a support, unless otherwise is stated.

Sample notation	Metal content, wt%			
	by loading		by elemental analysis	
	In	Co	In	Co
3In	3	0	3.2	0
1.5In	1.5	0	1.8	0
3In/3Co	3	3	3.1	3.0
3In/1.5Co	3	1.5	3.3	1.6
3In/0.3Co	3	0.3	3.2	0.3
1.5In/1.5Co	1.5	1.5	1.6	1.7
1.5In/0.9Co	1.5	0.9	1.7	1.0
1.5In/0.6Co	1.5	0.6	1.6	0.6
1.5In/0.3Co	1.5	0.3	1.6	0.4
1.5In/0.1Co	1.5	0.1	1.7	0.2
1.0In/0.5Co	1.0	0.5	1.2	0.5
0.7In/0.7Co	0.7	0.7	0.8	0.8
1.5In/0.3Co without Fe_3O_4	1.5	0.3	1.7	0.4
1.5In/0.3Co without PPP	1.5	0.3	1.6	0.4
3In/3Co without PPP	3	3	3.3	3.2

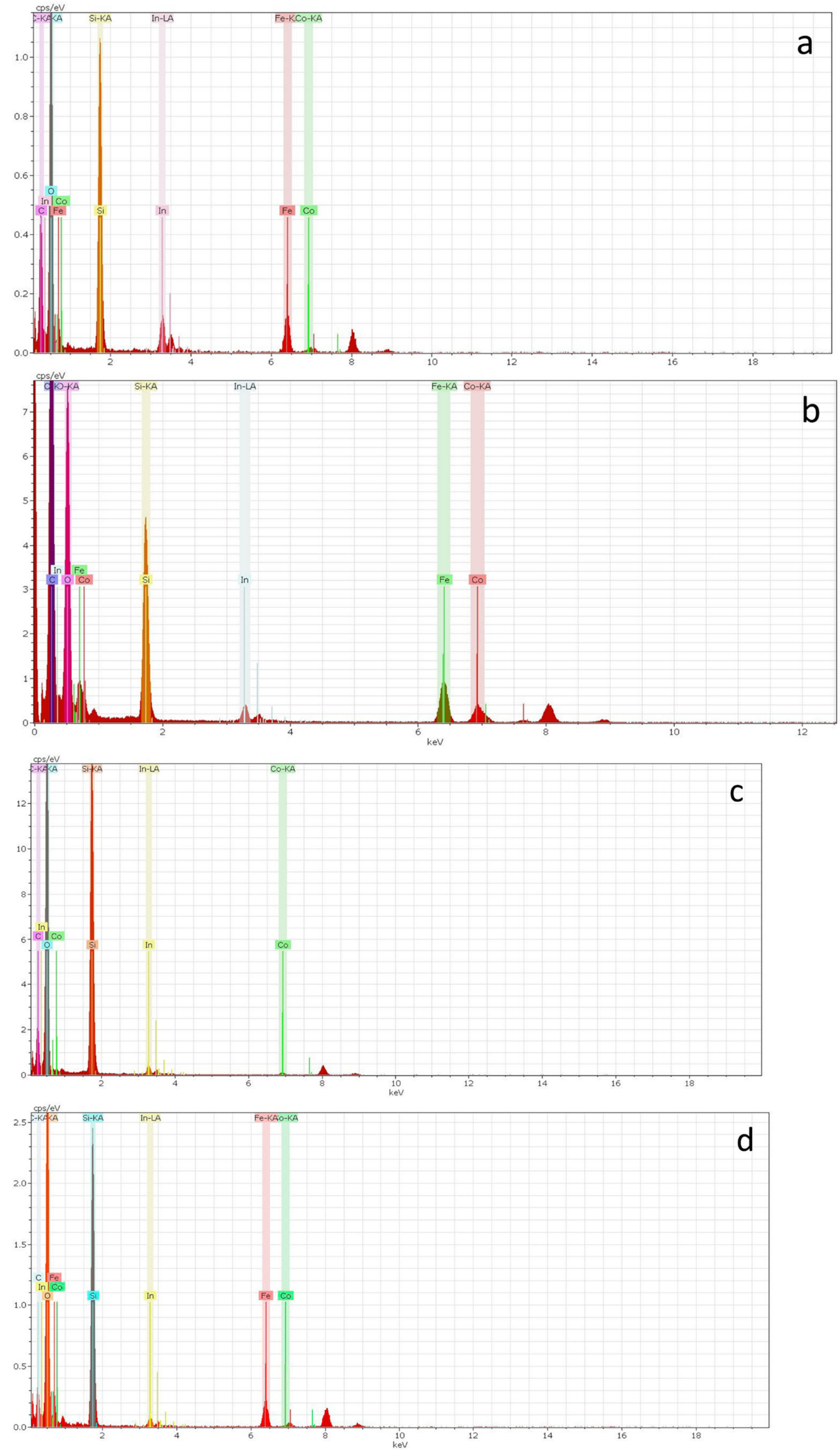

Figure S1. EDX spectra of 1.5In/0.3Co **(a)**, 3In/3Co **(b)**, 1.5In/0.3Co without Fe_3O_4 **(c)** and 1.5In/0.3Co without PPP **(d)**.

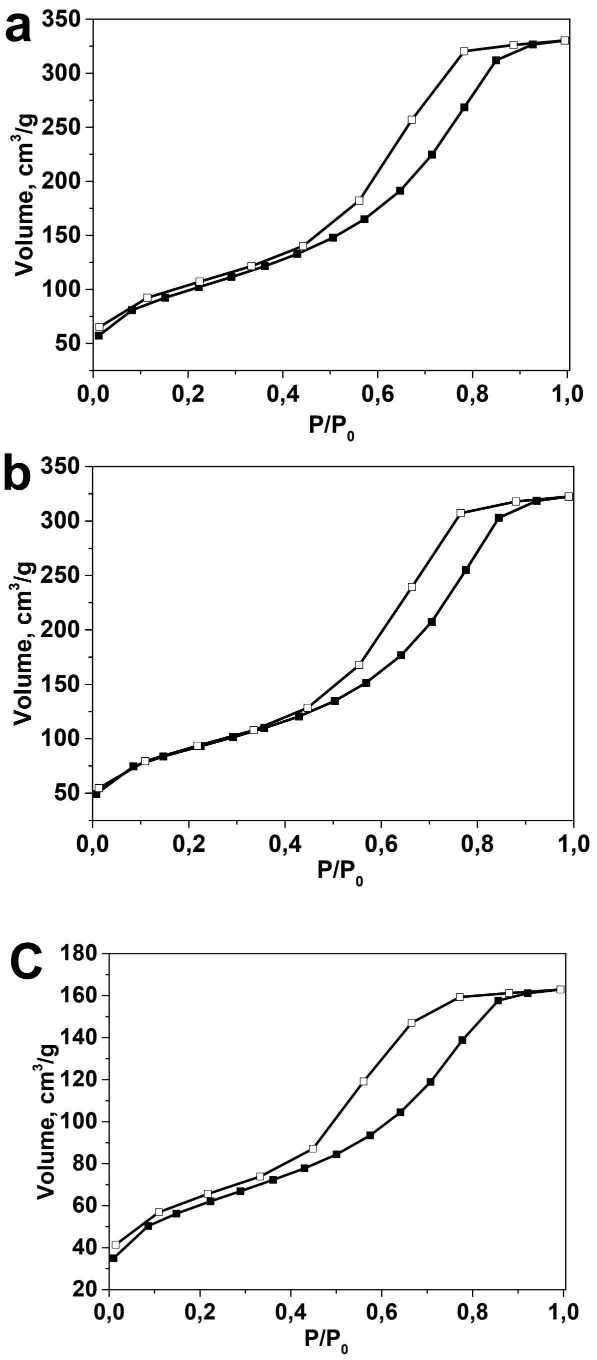

Figure S2. N_2 adsorption-desorption isotherms of 1.5In **(a)**, 1.5In/0.3Co **(b)** and 3In/3Co **(c)** catalysts.

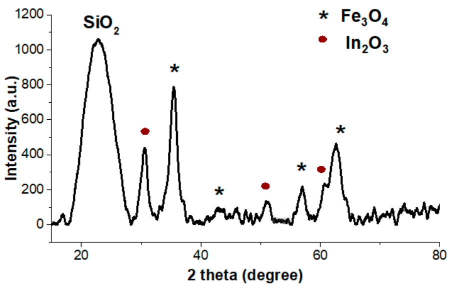

Figure S3. XRD pattern of 1.5In/0.3 Co without PPP.

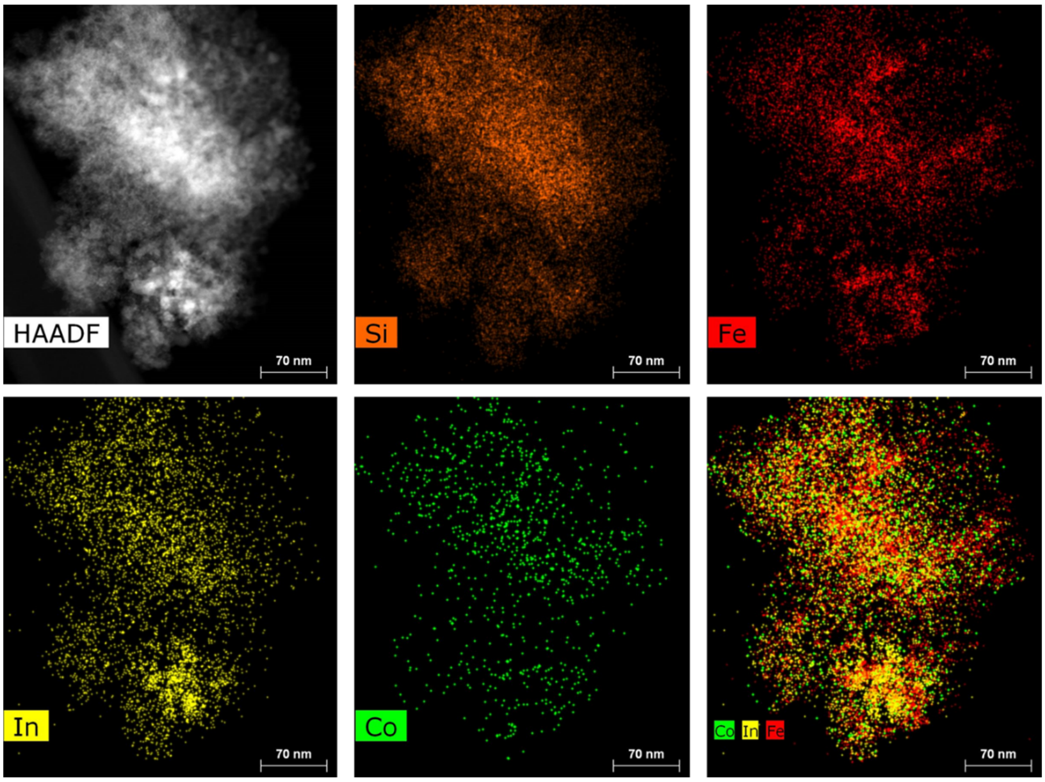

Figure S4. STEM dark field image and EDS maps of Si, Fe, In, Co and their superpositions of the 1.5In/0.3Co catalyst without the polymer layer.

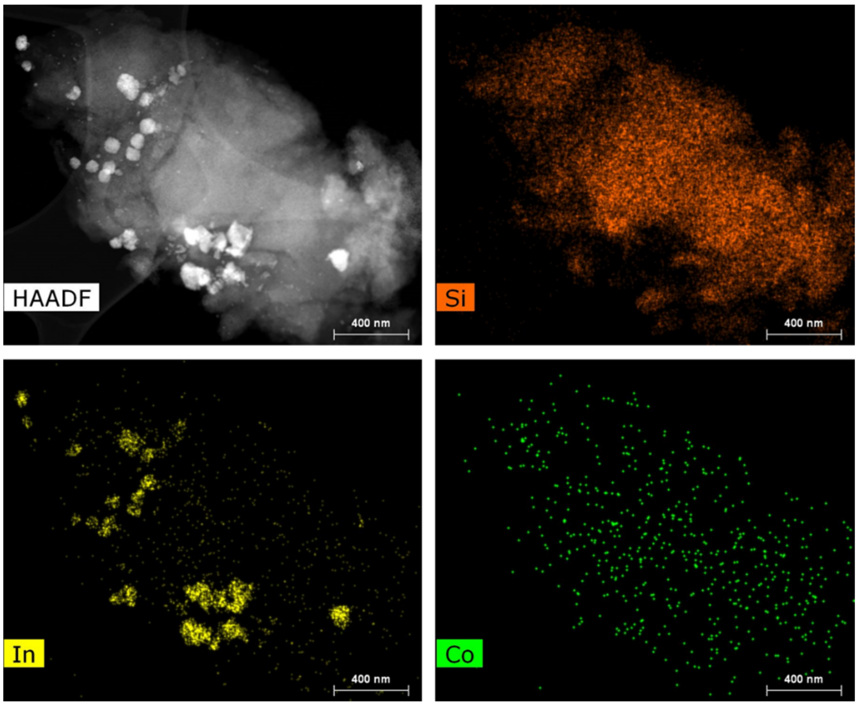

Figure S5. STEM dark field image and EDS maps of Si, In, Co and their superpositions of the 1.5In/0.3Co without magnetic NPs.

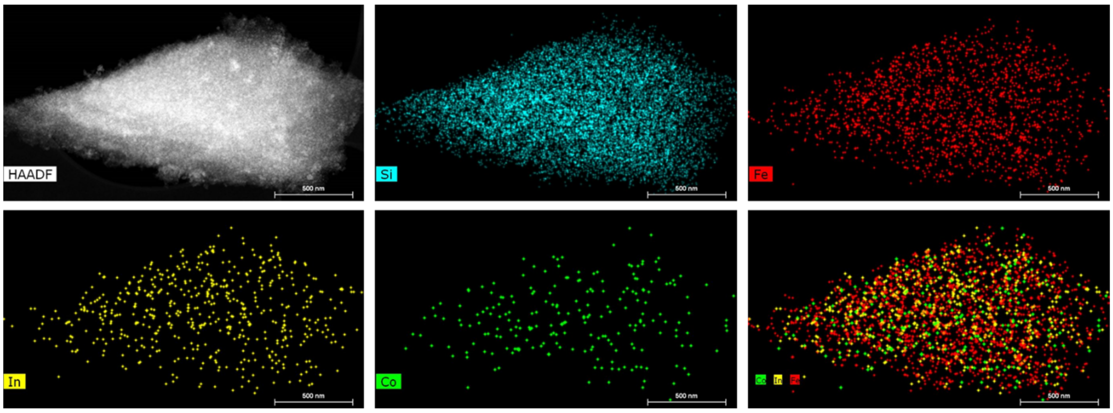

Figure S6. STEM dark field image and EDS maps of Si, Fe, In, Co and their superpositions of the 1.5In/0.3Co catalyst after the catalytic experiments.

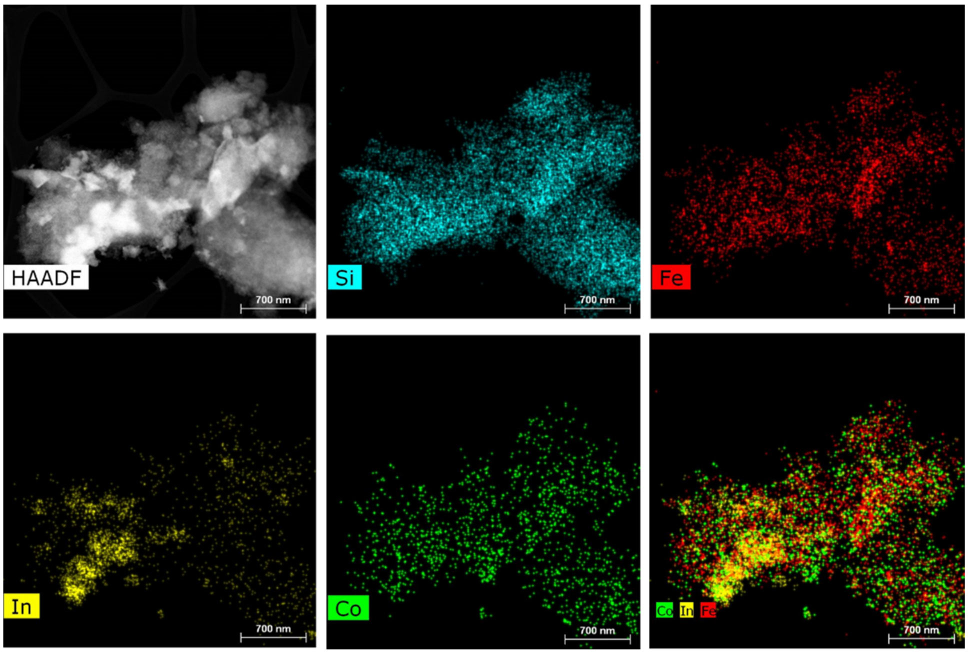

Figure S7. STEM dark field image and EDS maps of Si, Fe, In, Co and their superpositions of the 3In/3Co catalyst after the catalytic experiments.

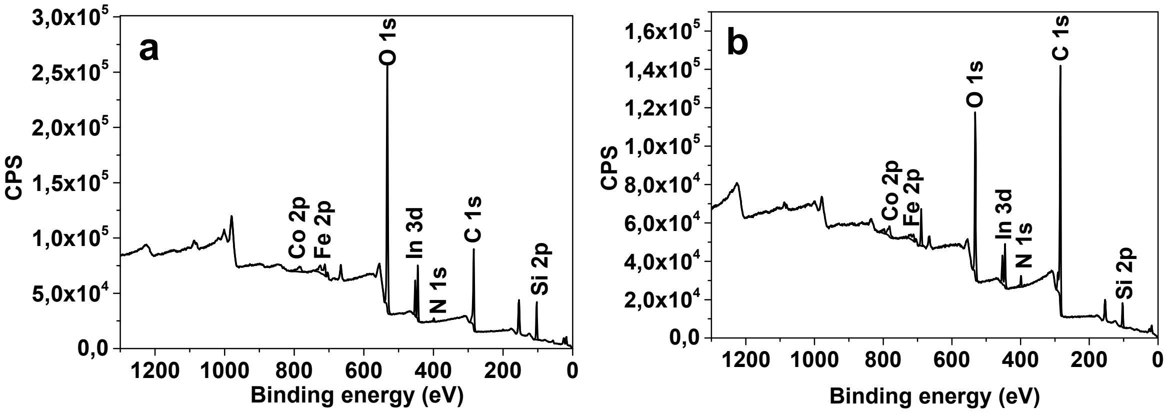

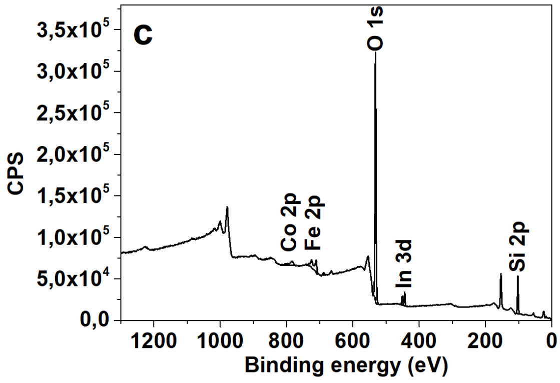

Figure S8. XPS survey spectra of 1.5In/0.3Co **(a)**, 3In/3Co **(b)** and 1.5In/0.3Co without PPP **(c)**.

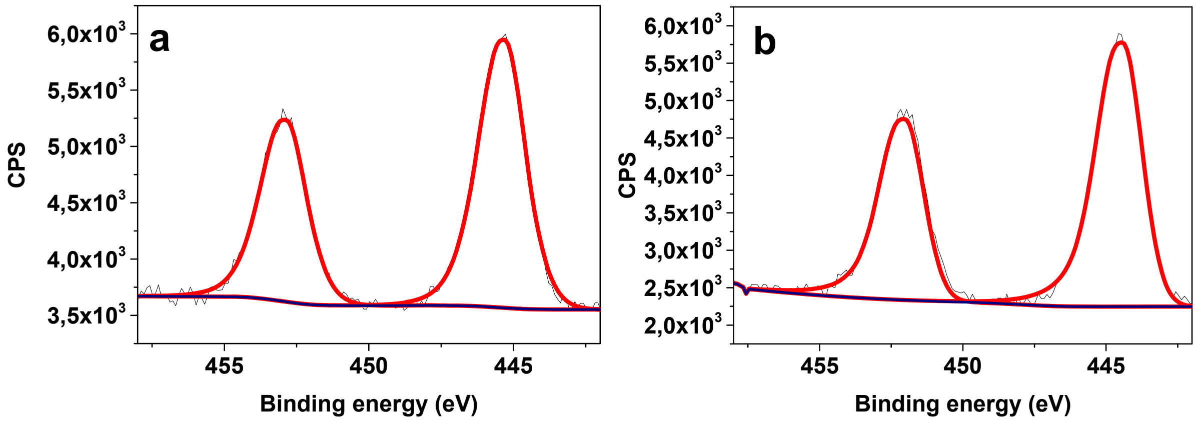

Figure S9. HR XPS spectra of In 3d of 3In/3Co catalyst **(a)** and 1.5In/0.3Co without PPP **(b)**.

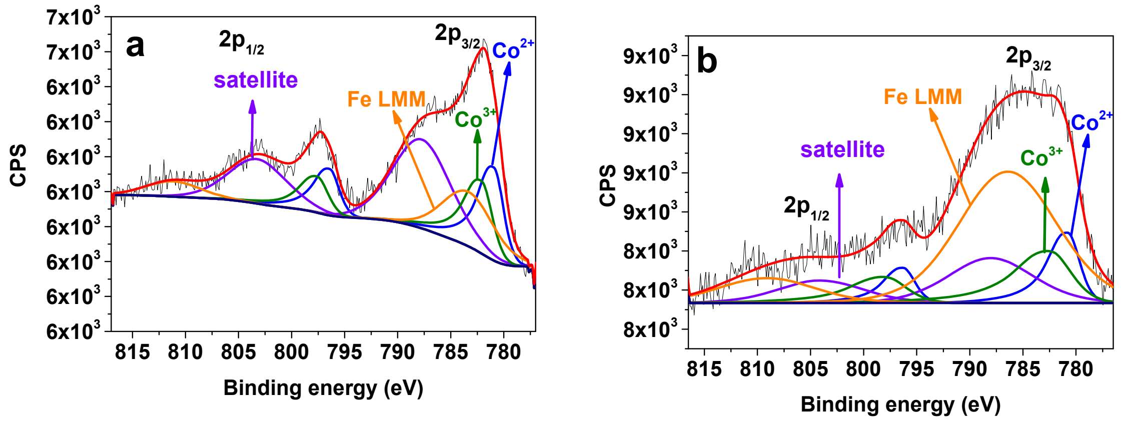

Figure S10. HR XPS spectra of Co 2p of 3In/3Co catalyst **(a)** and 1.5In/0.3Co without PPP **(b).**

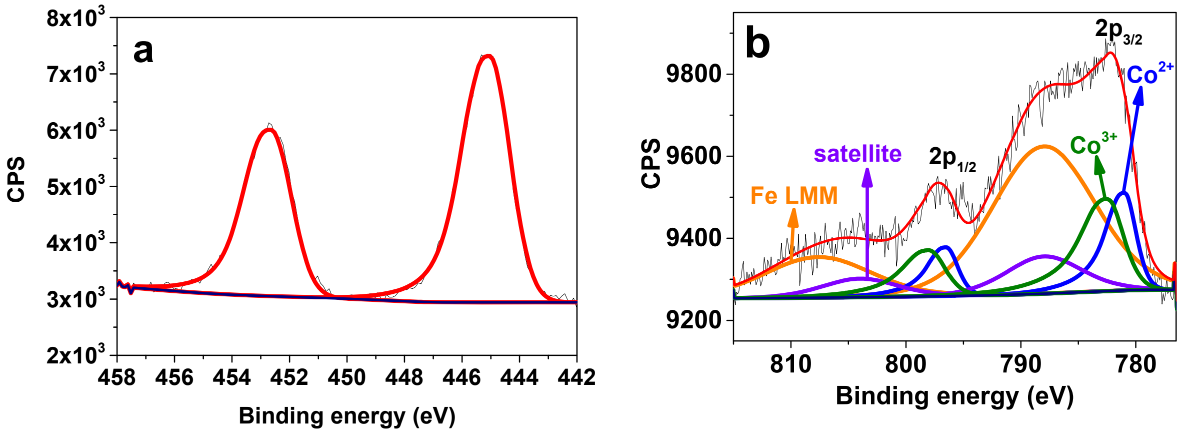

Figure S11. HR XPS spectra of In 3d **(a)** and Co 2p **(b)** of 1.5In/0.3Co after catalysis.

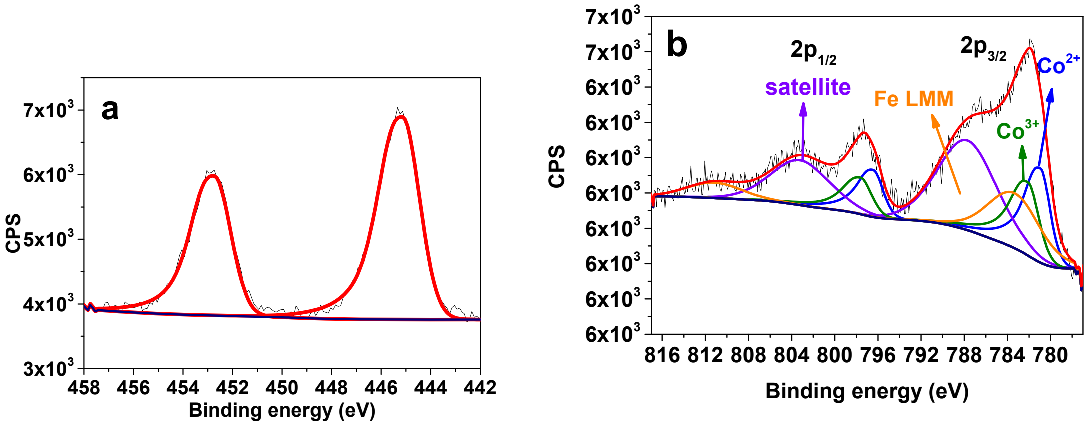

Figure S12. HR XPS spectra of In 3d **(a)** and Co 2p **(b)** of 3In/3Co after catalysis.

Table S2. Fitting parameters for HR XPS of Fe 2p of 1.5In/0.3Co before catalytic experiments.

	Pos.	FWHM	%Area
In $3p_{1/2}$	704.52	3.11	13.53
Fe $2p_{3/2}$ (Fe^{2+})	711.20	2.33	13.12
Fe $2p_{1/2}$ (Fe^{2+})	724.80	2.56	6.56
Fe $2p_{3/2}$ (Fe^{2+}) Satellite	720.83	8.00	14.99
Fe $2p_{1/2}$ (Fe^{2+}) Satellite	734.43	8.00	7.50
Fe $2p_{3/2}$ (Fe^{3+})	712.80	2.87	14.85
Fe $2p_{1/2}$ (Fe^{3+})	726.40	2.87	7.43
Co LMM	717.00	12.00	8.78
Fe $2p_{3/2}$ (Fe^{3+}) Satellite	715.32	3.93	8.83
Fe $2p_{1/2}$ (Fe^{3+}) Satellite	728.92	3.93	4.42

Table S3. Fitting parameters for HR XPS of Fe 2p of 1.5In/0.3Co after catalytic experiments.

	Pos.	FWHM	%Area
In $3p_{1/2}$	704.26	2.69	6.18
Fe $2p_{3/2}$ (Fe^{2+})	711.17	2.92	25.90
Fe $2p_{1/2}$ (Fe^{2+})	724.77	3.21	12.95
Fe $2p_{3/2}$ (Fe^{2+}) Satellite	717.96	8.06	14.42
Fe $2p_{1/2}$ (Fe^{2+}) Satellite	731.56	8.06	7.21
Fe $2p_{3/2}$ (Fe^{3+})	713.50	4.12	15.94
Fe $2p_{1/2}$ (Fe^{3+})	727.10	4.12	7.97

Co LMM	720.00	10.00	9.42

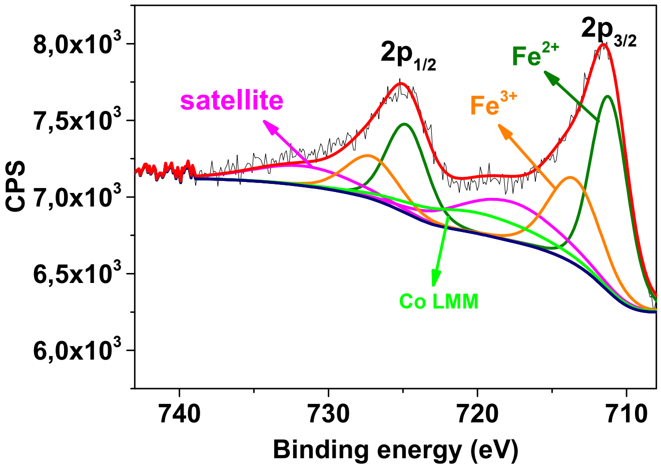

Figure S13. HR XPS spectra of Fe 2p of 1.5In/0.3Co after catalysis.

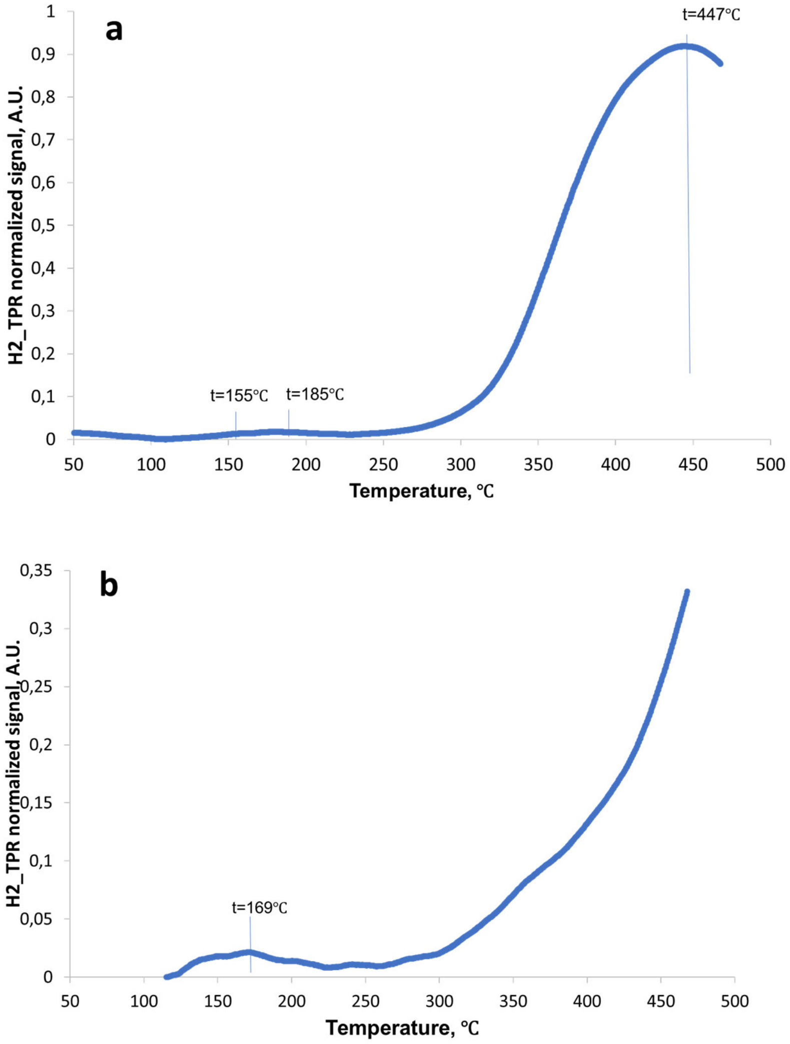

Figure S14. H_2 TPR profiles of 1.5In/0.3Co without PPP **(a)** and without Fe_3O_4 **(b)**.

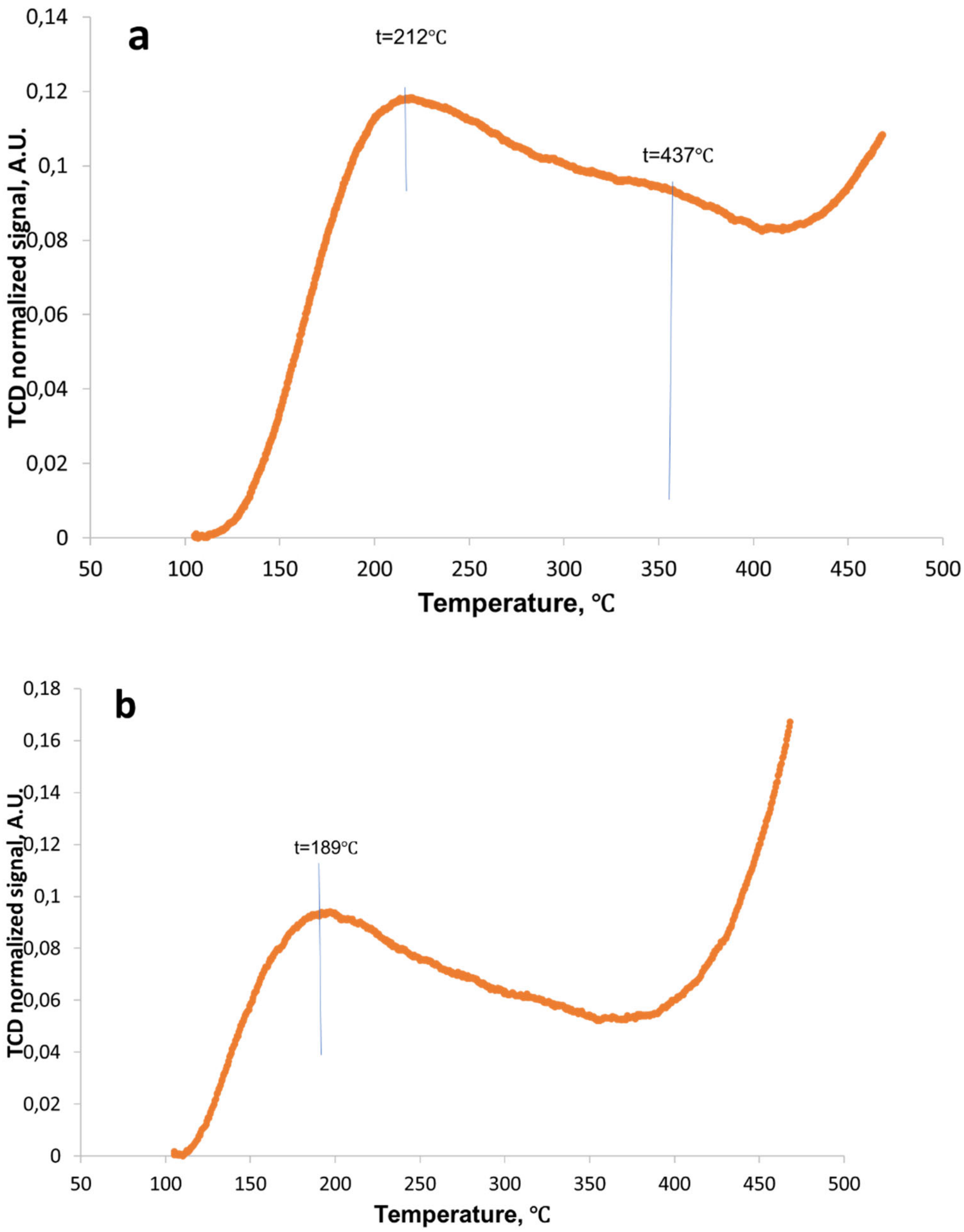

Figure S15. CO_2 TPD profiles of 1.5In/0.3Co without PPP **(a)** and without Fe_3O_4 **(b)**.
